# Strategic Management of Low Carbon Travel in Longevity Tourism Evidence from Thailand

**DOI:** 10.12688/f1000research.178615.2

**Published:** 2026-04-24

**Authors:** Warach Madhyamapurush

**Affiliations:** 1 University of Phayao, Mueang Phayao District, Phayao, Thailand

**Keywords:** Long-term tourism, emissions, carbon, transportation, lifestyle.

## Abstract

The increase in longevity tourism, driven by an aging populace and wellness travel, raises environmental issues, notably carbon emissions from travel. Thailand is becoming a key destination for this type of tourism, necessitating strategic frameworks to integrate low-carbon travel methods into its management. This research intends to evaluate the carbon footprint of longevity tourism travel patterns in Thailand and provide strategic management solutions to encourage low-carbon travel and improve long-term health and well-being results. A mixed-method approach was used to gather primary data via structured questionnaires and travel activity logs from 450 longevity tourists in selected Thai destinations, along with secondary data on emission factors from national energy and tourism databases. Carbon footprint assessment was conducted using an LCA-based carbon footprint analysis. Descriptive statistics and One-Way ANOVA in IBM SPSS (version 28) analyzed emissions variations across transportation modes, accommodation types, dietary choices, and activity patterns. Thematic analysis on qualitative responses highlighted common behavioral patterns and perceptions related to low-carbon travel practices. According to the LCA-based carbon footprint analysis, transportation (347 kg CO2e per capita) and lodging (160 kg CO2e) had the highest emissions, compared to lifestyle choices (42.5 kg CO2e) and strategic awareness factors (50 kg CO2e). Significant emission disparities among different travel modes, housing types, food patterns, and awareness levels were confirmed by one-way ANOVA results (p < 0.01). Longevity tourism in Thailand is made more sustainable by intentional low-carbon planning and optimized infrastructure, while extended stays and energy-efficient lodgings lower emissions.

## 1. Introduction

Several carbon-intensive sectors connected with the tourism development are the energy production, construction, transportation, and food supply systems. These areas support tourism mobility and cultural and natural attraction development and make tourism be essentially reliant on the energy and resources-intensive activities. It is common that the carbon footprint of tourism so called tourism carbon consumption effects both direct and indirect emissions through the transportation, accommodation and food consumed by the tourists in addition to the production and distribution of goods and services consumed by the tourists ( Yang et al., 2022; Zhong et al., 2025; Raihan, 2024b).

Longevity tourism can be defined as purposeful, and long-stay travel typically for individuals aged 45 years and above who are motivated to visit a destination for wellness improvement, health-seeking behavior, preventive care, or a preference for the retirement lifestyle, with a minimum stay of one month at the destination. This working definition distinguishes longevity tourism from traditional short-haul leisure tourism and permanent retirement migration. It is similar yet separate from more encompassing definitions of wellness tourism and long-stay tourism. Longevity tourism involves an extended engagement with a destination in terms of health, lifestyle, and social environment over a period of months, as opposed to wellness tourism, where such engagement occurs during short stays at spa or retreat facilities for just a few days. Unlike retirement migration, longevity tourism maintains the voluntary, temporary, and tourism-motivated nature of the visit, with participants primarily remaining residents of other locations ( Fakfare & Wattanacharoensil, 2024; Wang et al., 2023). Thailand has emerged as one of the top destinations to cater to this segment due to its internationally competitive healthcare system, wellness-focused culture, cost of living factors, and tropical climate, attracting both domestic Thai individuals aged 45 and above and international tourists seeking long-term wellness-driven experiences ( Janchai and Suvittawat, 2025).

Low-carbon tourism has originated as one of the important avenues towards realizing carbon neutrality in the tourism sector in response to the mounting environmental pressures. Low-carbon tourism aims at minimizing the emission of GHGs and the utilization of resources without jeopardizing the quality and economic feasibility of tourism. This approach has continued to attract more attention both in academia and the industry by combining environmental sustainability and tourism operations. It is particularly applicable to longevity tourism where prolonged periods of stay and activities that are focused on lifestyles would greatly contribute to cumulative carbon footprint of a destination, unless controlled ( Wang et al., 2023; Wan et al., 2025; Mao et al., 2022). This is because the need to embrace low-carbon tourism practices has been compounded by the fact that the world has become more exposed to climatic changes and growing carbon emissions, which pose a threat to tourism destinations. Tourism industry that had earlier been termed as being a low-impact industry or a smoke-free industry is being mentioned as one of the key contributors to carbon emissions to the planet and this has been catalysed largely by the transportation sector, accommodation, and activities, which make part of the lifestyle. The new sustainability issues are further aggravated by the fast growth of longevity tourism, which implies increased periods of stay and wellness tourism and prolonged patterns of consumption, which have increased per-capita emissions in the destinations ( Zhan et al., 2025; Hatamifar et al., 2025a, b). Transportation is the largest contributor to tourism-related emissions, with transportation providing over 75% of total tourism-related CO _2_ emission, especially by air and road transportation. Due to the strong impact of the transport options on the total tourism carbon footprint, awareness on tourist travel behavior and mobility patterns is important in finding some effective means of reducing the emission. The measurement of tourism-related CO _2_ emission is therefore a critical source of evidence to the formulation of low-carbon travel practices and strategic management interventions that could lessen the environmental effects and contribute to sustainable destination development ( Yan & Phucharoen, 2024; Wu et al., 2023). Longevity tourism presents health benefits through long-term stays and wellness lifestyles; however, they can significantly increase carbon emissions due to resource consumption and mobility. Implementing low-carbon travel frameworks that are integrated and behavior-sensitive can help mitigate these environmental impacts in longevity tourism destinations.

### 1.1 Research objective and contributions

The main aim of the research is to measure the effect of the longevity tourism travel trend in Thailand on the carbon emission. The following are some of its contributions:

➢ Provides an empirical, LCA of the carbon emissions associated with the Thailand longevity tourism travel patterns.
➢ Highlights low-carbon travel strategies to reduce per-capita emissions without compromising satisfaction among tourists, singling out housing and transportation as primary sources of emission.
➢ A sustainable long-term tourism framework with a combination of low-carbon planning, optimization of infrastructures, and behavioral interventions to the strategic management is proposed.

### 1.2 Research organization

The research is divided into six parts: Section 1 addresses the issue of the increased longevity tourism in Thailand and the carbon emission challenges. Section 2 analyses past studies on environmentally friendly tourism. Section 3 explains a mixed-method method of research, combining surveys of 450 tourists and the different forms of analysis. Section 4 gives the results of carbon emission and behavior pattern. Section 5 provides implications on sustainable tourism management. The conclusions and useful recommendations on future research are provided in section 6.

## 2. Related works

Naik et al. (2025) considered variables in determining the selection of eco-friendly tourist by the Indian tourists, emphasizing on the sustainability knowledge, awareness of the carbon footprint, and environmental issues. The research conducted a PLS-SEM analysis to explore the impact of sociodemographic factors and sustainability consciousness on tourism destinations. It found a positive correlation between environmental concern and sustainable travel intentions, with age moderating both the intention-behavior relationship and the link between sustainability consciousness and travel intention. However, the research focus on specific Indian locations can limit the generalizability of its findings. The techniques, important conclusions, and constraints pertinent to the development of sustainable tourism are highlighted in Table 1, which highlights recent research on how tourism, energy usage, and innovation affect carbon emissions.

**Table 1.** Questionnaire items on low-carbon travel practices in longevity tourism.

Variable	Question 1	Question 2
**Low-Carbon Travel Behavior**	**Q1:** Do you prefer using low-carbon transportation options (e.g., public transport, walking, or cycling) during your travels in Thailand?	**Q2:** Does awareness of environmental impact influence your choice of transportation during your trips?
**Sustainable Accommodation Choices**	**Q3:** Do you consider staying in energy-efficient or eco-certified accommodations during your trips?	**Q4:** Does the availability of sustainable lodging options encourage you to extend your stay or revisit the destination?
**Low-Carbon Lifestyle Adoption**	**Q5:** Do you prefer consuming local or plant-based meals to reduce your travel-related carbon footprint?	**Q6:** Do you participate in low-impact activities (e.g., walking tours, nature-based experiences) to minimize emissions during your visit?
**Strategic Awareness and Support**	**Q7:** Are you aware of renewable energy initiatives or sustainable infrastructure at tourism destinations in Thailand?	**Q8:** Does the presence of low-carbon infrastructure or policies influence your decision to visit or support a destination?

The recent studies emphasize the complicated correlation between tourism, carbon emissions, and low-carbon developmental policies. For example, Zhou et al. (2025) conducted surveys in Western Australia to determine how tourists make choices about transportation, with variables such as cost, time, and accessibility playing a significant role. However, this study is region-specific and may not be directly applicable to other geographical regions. Another study by Hatamifar et al. (2025a, b) assessed the carbon-neutral travel behavior of young Finnish tourists using questionnaires and found that positive results and social norms significantly influence sustainable behavior in this group. Unfortunately, this study was limited to Finland and may have limited relevance to other settings. Gallego et al. (2025) used composite indicators and carbon measures to evaluate the effects of focusing on low-emission tourism markets economically and ecologically. They concluded that focusing on low-emission markets is financially beneficial, but their study may oversimplify market processes and not consider how tourists will react to such changes. In their study, Pousa-Unanue et al. (2025) focused on the actions of urban tourists in Donostia/San Sebastian and evaluated environmental performance using spatiotemporal data and emissions. They found that nature tourists are the largest CO2 emitters, but this finding is limited by the study’s scope, which only covers one city.

A study conducted by Popović et al. (2025) used the greentripper tool to estimate the amount of carbon emitted by foreign tourists in Serbia, discovering that 80 percent of the carbon emissions were attributed to transportation and that the total carbon footprint was below what the rest of the world approximates. The results of this study, however, rely on national data and fail to consider regional or individual variations. Tsoggerel et al. (2025) used a panel dataset between the years 2000 and 2021 to analyze the implications of sustainable tourism and information and communication technology (ICT) on the quality of the environment in China in terms of its asymmetry. They found a trend with tourism reducing emissions at low, but also high quantiles, but restricting the generalizability of their findings to other countries since the study is centered on China. Over a sample of econometric methods, Raihan (2024a) evaluated the effects of tourism and energy-economic variables on emission levels in Malaysia, thus concluding that tourism and the use of fossil fuels increment production of emissions whereas the use of renewable energy sources reduces them. Nonetheless, geographic or environmental differences that could affect such findings were not taken into consideration in this study. The study by Voumik et al. (2024) looking at tourism’s contribution to CO2 emissions in 40 Asian countries revealed that tourism and renewable energy projects reduce carbon emissions, whereas economic growth and energy consumption produce the opposite effect. However, their conclusions fail to reflect all the regional differences and policymaking.

In a study by Yue et al. (2021) on green innovation and tourism in Thailand, it was found that the two industries have the potential to reduce CO _2_ emissions. However, the study has limitations due to its focus on sector-specific and local factors that may not be universal. In a study by Yue et al. (2025) on the impact of Low Carbon City Policies (LCCPs) on urban tourism growth in China, it was discovered that LCCPs effectively boost tourism receipts in urban areas by stimulating markets and enhancing the ecological environment, but the experiments were limited to Chinese cities. Zhao et al. (2024) investigated the effects of tourism development on carbon emission efficiency in 31 cities in China, finding that tourism has a positive influence on efficiency while foreign direct investment (FDI) has a negative influence. However, the city-specific focus of this research may overlook external factors that could impact the results. In a study by Janchai and Suvittawat (2025), surveys and structural equation modeling were used to assess tourists’ preferences for low-carbon destinations, revealing that destination characteristics and marketing activities significantly influence tourists’ perceptions and decision-making. Nevertheless, the reliance on self-reported data and the focus on a single location raise questions about the generalizability of these results.

In addition, a quantitative assessment of the CO _2_ emissions of the Chinese tourism industry was conducted by Chen et al. (2022). They found that the increase in emissions did not correspond to a decrease in intensity, but rather significant regional trends. However, the aggregated information method can mask information at the city level. Similarly, Tong et al. (2022) demonstrated the role of tourism in carbon drawdown in 92 Chinese cities. They found that tourism produced a net positive effect on carbon dioxide emissions, but the adoption of structural equation modeling did not allow them to examine non-linear relations and spatial transformations. Khiaolek et al. (2025) also revealed the potential for greenhouse gas (GHG) reduction in tourist destinations like Chiang Mai, Thailand. This was acknowledged through surveys and gap analysis, providing an overall greenhouse gas reduction potential of 15,304.72 tCO2eq to be achieved in Chiang Mai, especially through the use of solar energy. However, since they focused on one province, their findings may be limited in wider applicability. Fakfare and Wattanacharoensil (2024) suggested specific low-carbon tourist segments using cluster analysis and a survey, but their study was restricted to domestic tourists, limiting the applicability of the findings to international settings. In a hybrid life cycle assessment, Platts et al. (2023) found the carbon footprint of visitors to 16 UNESCO World Heritage Sites and produced trip-wise footprint data that may be more useful in communicating climate impact. However, their results may not be widely applicable to a broader range of tourism sites due to their UNESCO-centric focus. Finally, Guo et al. (2025) interviewed tourists in Macao about their carbon awareness using a special questionnaire. They found that a significant portion of tourists are at the initial level of carbon awareness, with their Carbon Action activities contributing to some of them. The results, however, are limited by the localized nature of the study and would require cross-cultural validation. All of these studies highlight the complex relationship between tourism and carbon emissions, showing unique patterns and issues, as well as the need for further investigation in various settings to develop successful low-carbon strategies in the tourism industry ( Nathaniel et al., 2023).

### 2.1 Research gap

In the knowledge of the low-carbon travel practices and tourism carbon emission has some gaps. Some of them are the reliance on studies that focus to specific regions or countries, and this can restrict the extrapolation of findings to other tourist destinations or tourist settings ( Naik et al., 2025). Moreover, certain methods simplify complicated tourism systems and do not consider how tourists react to carbon-oriented efforts ( Gallego et al., 2025). The results cannot apply to other nations or situations because they are solely based on China ( Tsoggerel et al., 2025).

This study identifies four key gaps in the current literature on longevity tourism. Firstly, there is a lack of studies conducting a per-capita carbon footprint analysis using a lifecycle assessment specifically for longevity tourism. Secondly, existing behavior-sensitive frameworks for low-carbon tourism have not been applied to longevity tourists. Thirdly, there is a gap in destination-level carbon accounting for the longevity tourism segment in Thailand. Lastly, there is a lack of integration of quantitative emission estimation with qualitative analysis of tourist behavior and perceptions in the context of longevity tourism in Asia. This study aims to address these gaps by providing an integrated, mixed-method, LCA-based carbon footprint assessment of longevity tourism in selected Thai destinations and proposing a behavior-sensitive strategic framework for low-carbon longevity tourism management.

## 3. Research design

**Figure 1.**
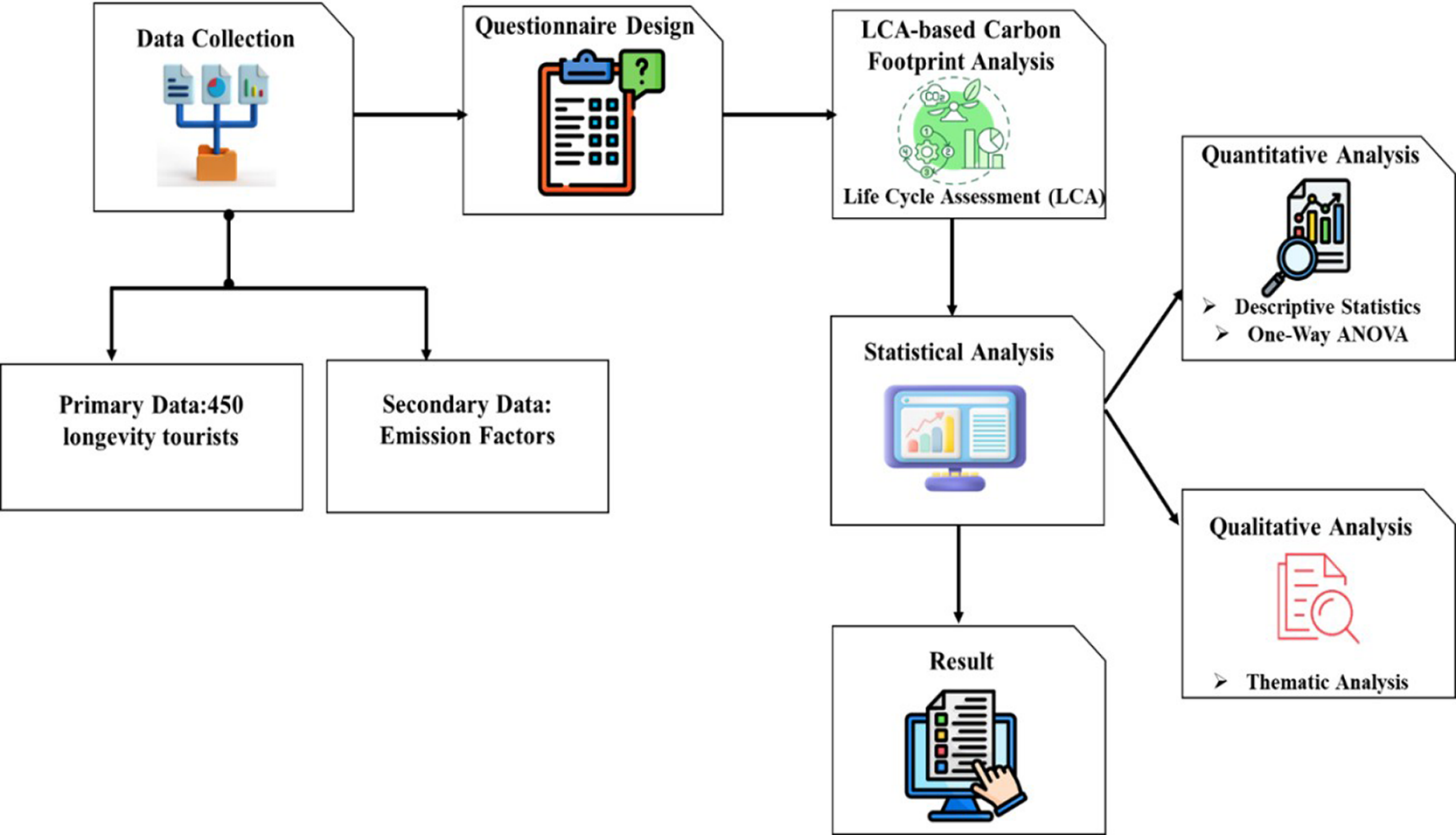
Research framework for assessing low-carbon practices and carbon footprint in Thai Longevity Tourism.

The research evaluates the long-term tourism industry carbon footprint of Thailand using a mixed-method approach. The structured survey and travel activity records of longevity tourists were used to gather primary data, whereas national energy and tourism databases were used to obtain secondary data on the factors of emissions. The carbon footprint analysis of the carbon emissions was done through an LCA and the analysis of the changes in lodging, transportation, food, and the activities was done using one-way ANOVA and descriptive statistics. Figure 1 depicts the general research framework for evaluating low-carbon behaviors and carbon footprint in Thai longevity tourism.

### 3.1 Data collection

Data were collected across five purposively selected Thai destinations — Chiang Mai, Hua Hin, Pattaya, Bangkok, and Phuket — which collectively represent the principal geographic, infrastructural, and market-type clusters of longevity tourism in Thailand, encompassing urban medical-wellness hubs, coastal retirement enclaves, and international resort-wellness destinations.

Structured questionnaires and travel activity logs were used to collect primary data on the housing, transportation, food preferences, and other activities of the 450 longevity tourists in certain areas in Thai locations ( Madhyamapurush, 2026). The carbon footprint research was supported using secondary data on emission factors obtained through the national energy and tourism databases. The use of both primary and secondary information provides the right solution to the monitoring of the travel trends and emission levels.

For inclusion in the study, a longevity tourist was operationally defined as an individual aged 45 years or above who was residing in Thailand for a minimum of 30 consecutive days and whose primary travel motivation was wellness enhancement, health-seeking, preventive healthcare, or retirement lifestyle, consistent with the conceptual definition.

***3.1.1 Sampling Strategy and Recruitment***

Participants were recruited using a purposive convenience sampling strategy at five longevity tourism destinations in Thailand selected to represent the geographic and infrastructural diversity of the longevity tourism market: Chiang Mai (northern wellness and cultural hub), Hua Hin (coastal retirement and wellness enclave), Pattaya (coastal long-stay zone), Bangkok (urban medical and wellness destination), and Phuket (international resort and wellness island). These sites were selected because they collectively account for the largest concentrations of long-stay wellness visitors in Thailand and offer a range of accommodation types, healthcare facilities, and lifestyle environments relevant to longevity tourists. Participant recruitment was conducted at wellness centers, health resorts, eco-certified hotels, international hospitals offering wellness programs, and expatriate community centers at each site over a ten-week data collection period.

To be included in the study, participants were required to meet the operational definition of longevity tourist: aged 45 years or above, currently staying in Thailand for a minimum of 30 consecutive days, and traveling primarily for wellness, health, preventive care, or retirement lifestyle purposes. Participants whose primary reason for travel was business, conventional leisure, or medical treatment only (without a wellness or lifestyle component) were excluded. Potential participants were first screened using a two-question eligibility filter administered verbally by the research team before questionnaire administration. A total of 510 individuals were approached; 450 met the inclusion criteria and provided completed, usable questionnaires, yielding a response rate of 88.2%.

### 3.2 Questionnaire design

A survey questionnaire was prepared to capture the data on the demographics of participants, the patterns of travel, and low-carbon behavior amid brand longevity tourism in Thailand depicted in Table 1. To measure the sustainability of the travel practices, the tool provided questions regarding accommodation, food, destination, mode of transport and knowledge of the renewable energy programmes. The questionnaire involved multiple-choice, open-ended and Likert-scale questions to understand the adoption of low-carbon activities and the impacts on the decisions of visitors.

Questions Q1 to Q8 each employed a five-point Likert-type response scale anchored from 1 (Strongly Disagree /Never) to 5 (Strongly Agree/Always). The questionnaire also included two open-ended qualitative questions — ‘What factors most influence your transportation choices during your stay?’ and ‘What changes in destination infrastructure or policy would most encourage you to adopt lower-carbon behaviors?’ — the responses to which were subject to thematic analysis. Prior to field deployment, the questionnaire underwent a two-stage validation process: (i) content validity was assessed through expert review by three academics with expertise in tourism sustainability and carbon footprint research, resulting in minor wording clarifications for Q3 and Q7; and (ii) a pilot test with 30 longevity tourists (excluded from the main analysis) was used to assess instrument reliability. Cronbach’s alpha values from the pilot indicated satisfactory internal consistency for each of the four construct domains: Low-Carbon Travel Behavior (α = .79), Sustainable Accommodation Choices (α = .76), Low-Carbon Lifestyle Adoption (α = .81), and Strategic Awareness and Support (α = .74). For the main analysis, composite scores for each domain were computed as the arithmetic mean of the two constituent Likert items, yielding a continuous 1–5 scale per domain.

### 3.3 Carbon footprint assessment

To assess the emissions of GHGs related to the longevity tourism travel patterns in Thailand, the research employs the carbon footprint analysis of LCA method. It measures the travel, housing, food, and tourism emissions based on primary trip data and on a set of predetermined emission parameters. The evaluation makes it possible to compare per-capita emissions when traveling a certain distance and performing a specific behavior. This solution help to identify the low-carbon travel solutions that could be effective without negatively affecting the well-being of visitors.

***3.3.1 Lifecycle Assessment (LCA) for Carbon Footprint Evaluation and Low-Carbon Strategy in Longevity Tourism***

This study uses a bottom-up LCA approach to calculate greenhouse gas emissions per capita for longevity tourism in Thailand. The analysis includes emissions from transportation, accommodation, dietary consumption, and recreational activities. Emission factors are sourced from reputable organizations, and key factors used in calculations are provided. Per-capita emissions are calculated for each domain, considering travel distances, accommodation stays, dietary choices, and activity-specific emissions. Assumptions and sensitivity analyses are conducted to assess the impact of variations in key factors on emission estimates.

### 3.4 Statistical analysis

Variations in carbon emissions across various travel patterns, dwelling types, means of transportation, and lifestyle activities of longevity tourists in Thailand were assessed using statistical analysis. The emission data was initially summarized using descriptive statistics in IBM SPSS (version 28), and any significant group differences were subsequently examined using One-Way ANOVA. The research employs a quantitative approach to identify major emission sources and assess low-carbon travel options. It includes thematic analysis of qualitative data from questionnaires and trip diaries, revealing behaviors and attitudes linked to low-carbon travel. This integrated analysis offers key insights into visitor behavior and travel patterns, contributing to the management of sustainable longevity tourism in Thailand.

The four key constructs — Low-Carbon Travel Behavior, Sustainable Accommodation Choices, Low-Carbon Lifestyle Adoption, and Strategic Awareness and Support — were operationalized at two levels for the analyses. At the behavioral level, each construct was represented by the per-capita carbon emission estimate (in kg CO _2_e) derived from the LCA calculation applicable to that domain, as described in Section 3.3. At the attitudinal level, each construct was represented by the composite mean score of its two Likert questionnaire items (Q1–Q2, Q3–Q4, Q5–Q6, Q7–Q8 respectively). For the One-Way ANOVA analyses, participants were grouped by the discrete travel behavior sub-categories reported in their activity logs (e.g., air travel vs. private vehicle vs. public transport for the transportation domain; hotel/resort vs. serviced apartment vs. eco-certified hotel for the accommodation domain), and the dependent variable in each ANOVA was the per-capita kg CO _2_e emission estimate for that domain. This approach directly links the questionnaire-derived sub-group classifications to LCA-derived emission outcomes, enabling statistically grounded comparison of emission levels across behavioral sub-groups.

➢ **Descriptive statistics**

Descriptive statistics were applied to the analysis of the travel patterns and carbon emissions of longevity tourists in Thailand and the patterns of transportation, accommodation, food, and activities were found. The information can help policy makers and tourism operators to determine the sources of high pollution and what low-carbon options are possible. The median offers a realistic view of typical travel behavior, while the mean indicates average emissions per traveller. Additionally, standard deviation highlights variances in hotel and transport preferences among travellers.

➢ **One-Way ANOVA**

ANOVA is a statistical procedure used to compare means of a continuous response variable of an analysis across multiple groups characterised by discrete variables. It ascertains the statistical significance of the differences in the means of the groups by the comparison of the intergroup variance with the intragroup variance. When the population means are more than two, the F-statistic is the test statistic. This research uses one-way ANOVA, to determine whether the means of transportation, accommodation facilities, and activities of longevity tourists in Thailand have significant impacts in their carbon emission.

Before interpretation, the following ANOVA assumptions were verified for each model: (i) normality of the dependent variable within each group was assessed using Q-Q plots and Shapiro-Wilk tests; (ii) homogeneity of variance across groups was assessed using Levene’s test. In models where Levene’s test indicated significant heteroscedasticity (p < .05), Welch’s robust one-way ANOVA was substituted for the standard F-test, and Games-Howell post-hoc comparisons were applied in place of Tukey HSD.

➢ **Thematic analysis**

The research employs the thematic analysis that systematically determines and rates recurrent themes in the qualitative answers of the longevity tourists and stakeholders. It seeks to identify some of the major themes in terms of travel patterns, hotel preference, activities, and low-carbon traveling attitudes. The approach adds to the existing knowledge of the impact of passenger-choice on carbon emissions, with a complementary role to quantitative data, and it can aid in the construction of low-carbon tourist policy in Thailand.

Thematic analysis was conducted on qualitative data obtained from two sources: (i) written responses to the two open-ended questionnaire items, and (ii) narrative entries from the travel activity logs in which participants described their in-destination behavior and motivations. Thematic analysis followed Braun and Clarke’s (2006) six-phase reflexive framework: familiarization with the data, generation of initial codes, searching for themes, reviewing themes, defining and naming themes, and producing the report. The analysis was conducted inductively in the first instance, with no pre-specified coding framework, to allow themes to emerge from participants’ own language and reasoning.

**Ethical considerations**

The research described in this article was reviewed and approved by the University of Phayao Human Ethics Committee, Thailand. The approval reference number is **HREC-HSS 2.2/175/68**. All procedures performed in studies involving human participants were in accordance with the ethical standards of the institutional and/or national research committee and with the 1964 Helsinki declaration and its later amendments or comparable ethical standards. Informed consent was obtained from all individual participants included in the study. Informed consent, in written form, was obtained from all individual participants included in the study.

## 4. Result

Research of the travel preferences of longevity tourists found out that most of them liked local means of transport and mid-range and energy efficient accommodation. The descriptive statistics and One-Way ANOVA indicates that the nature of lodging and mode of transport are the two most important factors that lead to carbon emission. Prolonged occupancy paired with eating and exercising options that are sustainable can reduce the emission per capita to a considerable degree. The thematic study indicates that low-carbon behaviors such as environmentally-friendly hotel, environmentally-friendly transportation preferences, and environmentally-friendly lifestyle experiences are taken by longevity tourists. These behaviors are all helped by infrastructure, policy assistance, renewable energy experiences and eco-awareness. These results show that deliberate low-carbon travel methods can successfully reduce the environmental impact without affecting the satisfaction of tourists.

### 4.1 Demographic distribution

**Figure 2.**
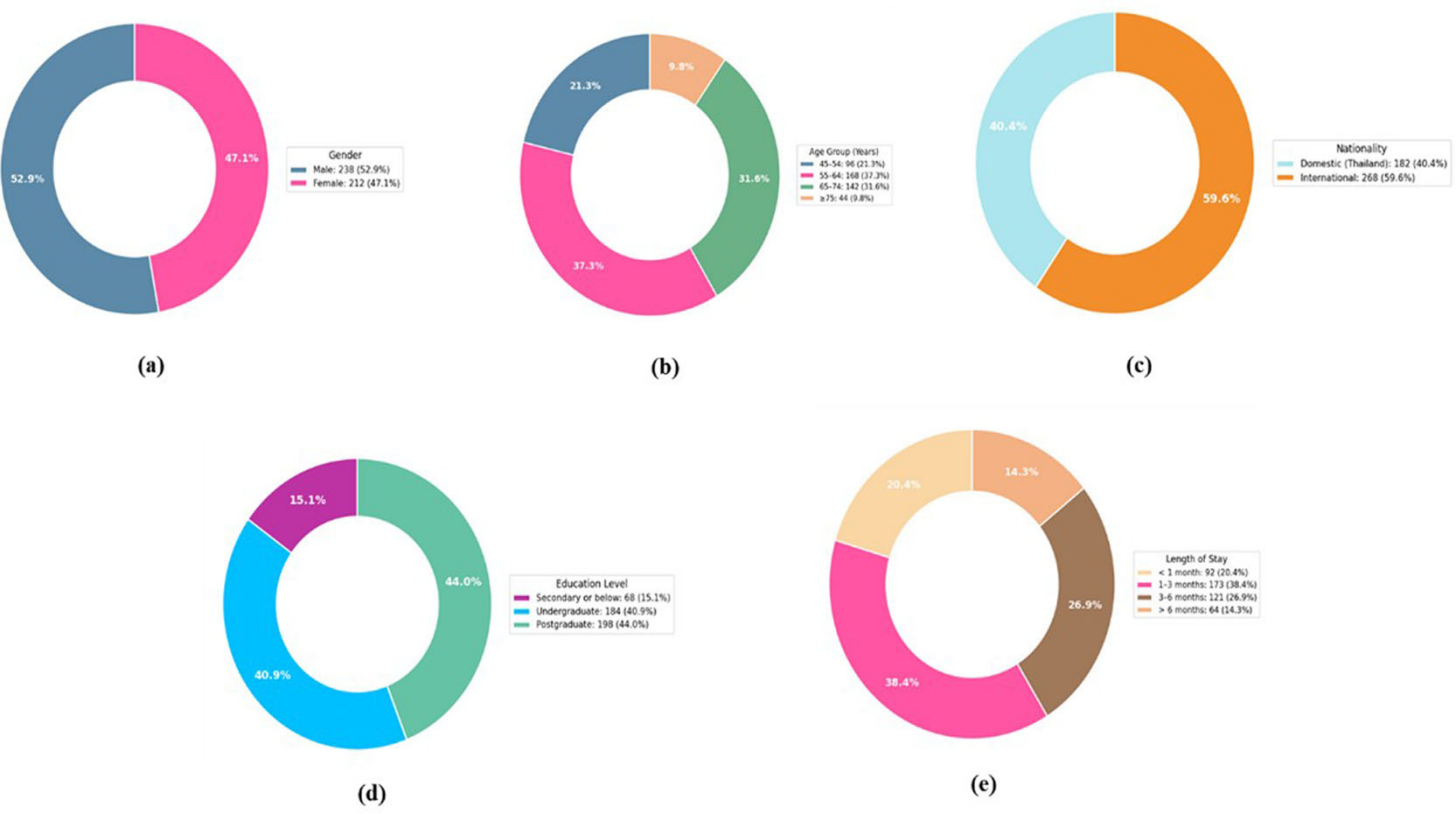
Demographic characteristics of longevity tourism respondents: (a) Gender, (b) Age Group, (c) Nationality, (d) Education Level, (e) Length of Stay.

The demographic distribution of the 450 responses is shown in Table 2, which reveals a balanced gender distribution with a small male predominance. Most longevity travellers are 55 years of age or older due to the senior-focused nature of long-term wellness travel. The fact that foreigners make up most visitors highlights Thailand’s allure as a long-term tourism destination. Long stays and wellness-focused travel choices are encouraged by the sample’s high level of education and middle-class to upper-class demographics. A significant portion of travellers stay longer than a month to assess carbon emissions and low-carbon travel activities. The demographics of longevity tourism respondents, including (a) gender distribution, (b) age group distribution, (c) nationality composition, (d) education level, and (e) duration of stay, are shown in Figure 2.

**Table 2.** Demographic distribution of longevity tourists (n = 450).

Demographic variable	Category	*Frequency* ( *n*)	*Percentage* (%)
**Gender**	Male	238	52.9
	Female	212	47.1
**Age Group (Years)**	45–54	96	21.3
	55–64	168	37.3
	65–74	142	31.6
	≥75	44	9.8
**Nationality**	Domestic (Thailand)	182	40.4
	International	268	59.6
**Education Level**	Secondary or below	68	15.1
	Undergraduate	184	40.9
	Postgraduate	198	44.0
**Length of Stay**	< 1 month	92	20.4
	1–3 months	173	38.4
	3–6 months	121	26.9
	> 6 months	64	14.3

### 4.2 LCA-based carbon footprint analysis

**Figure 3.**
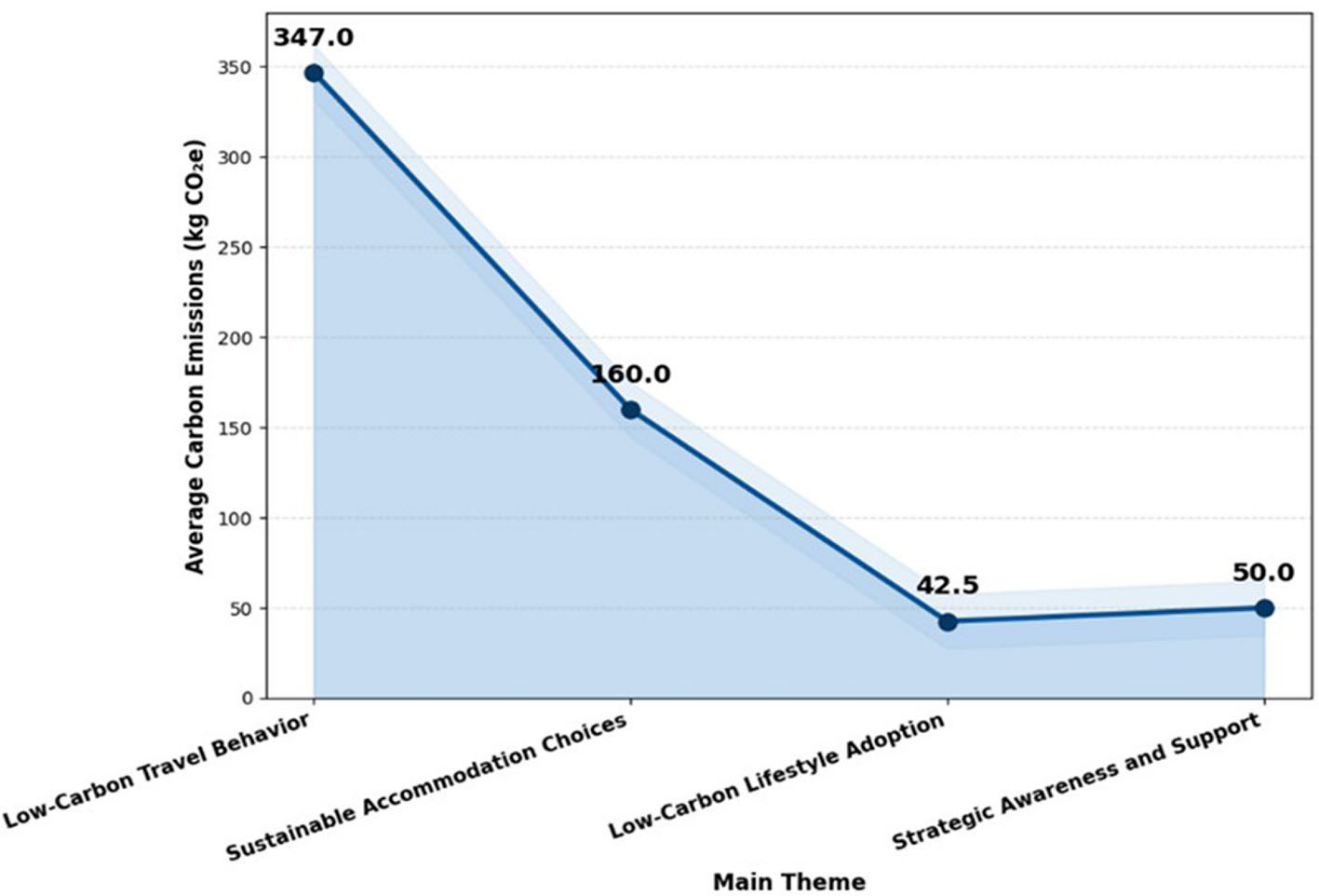
LCA-based comparison of carbon emission drivers in longevity tourism.

According to the LCA-based carbon footprint analysis, lifestyle decisions, activities, and strategic awareness have less of an impact on emissions in longevity tourism than transportation and lodging shown in Table 3 and Figure 3. The adoption of renewable energy, energy-efficient accommodation, and sustainable transportation are only a few of the important areas where low-carbon interventions can successfully lower the overall carbon footprint when emissions are categorized by major themes.

**Table 3.** LCA-based carbon footprint of longevity tourism activities in Thailand (per capita, *kg* *CO*_2_ *e*).

Main theme	Category	Average carbon emissions ( *kg* *CO*_2_ *e*)
**Low-Carbon Travel Behavior**	Transportation	347
**Sustainable Accommodation Choices**	Accommodation	160
**Low-Carbon Lifestyle Adoption**	Diet & Activities	42.5
**Strategic Awareness and Support**	Renewable Energy & Infrastructure	50

### 4.3 Descriptive statistics

The descriptive statistics in Table 4 indicates that Low-Carbon Travel Behavior and Sustainable Accommodation Choices significantly contribute to per-capita carbon emissions in longevity tourism. The focus on low-carbon lifestyle, knowledge and assistance, can help reverse the emissions, by reducing the effects of activities with low impact, the choice of food, and the awareness of renewable energy. These statistics highlight the main areas to follow the low-carbon strategies in Thai longevity tourism.

**Table 4.** Descriptive statistics of key low-carbon travel variables (n = 450).

Variable	Mean emissions ( *kg* *CO*_2_ *e*)	Standard deviation	Minimum	Maximum
Low-Carbon Travel Behavior	347	182.5	0	1,320
Sustainable Accommodation Choices	160	65.3	50	220
Low-Carbon Lifestyle Adoption	42.5	18.7	15	60
Strategic Awareness and Support	50	20.4	0	90

Note. Mean emissions and standard deviations (SD) are reported per capita (kg CO _2_e). The 95% confidence intervals for mean emissions are: Low-Carbon Travel Behavior: [330.1, 363.9]; Sustainable Accommodation Choices: [153.9, 166.1]; Low-Carbon Lifestyle Adoption: [40.8, 44.2]; Strategic Awareness and Support: [48.1, 51.9]. The high SD for Low-Carbon Travel Behavior (182.5) reflects the substantial variability in transport mode choice, particularly the contrast between air travelers (mean 950 kg CO _2_e) and public transport users (mean 90 kg CO _2_e).

The high standard deviation for Low-Carbon Travel Behavior (SD = 182.5) reflects the wide dispersion in transport mode emissions — primarily the contrast between air travelers and public transport users — and underscores the importance of transport mode as the single largest and most variable source of per-capita emissions in longevity tourism.

### 4.4 One-Way ANOVA

**Figure 4.**
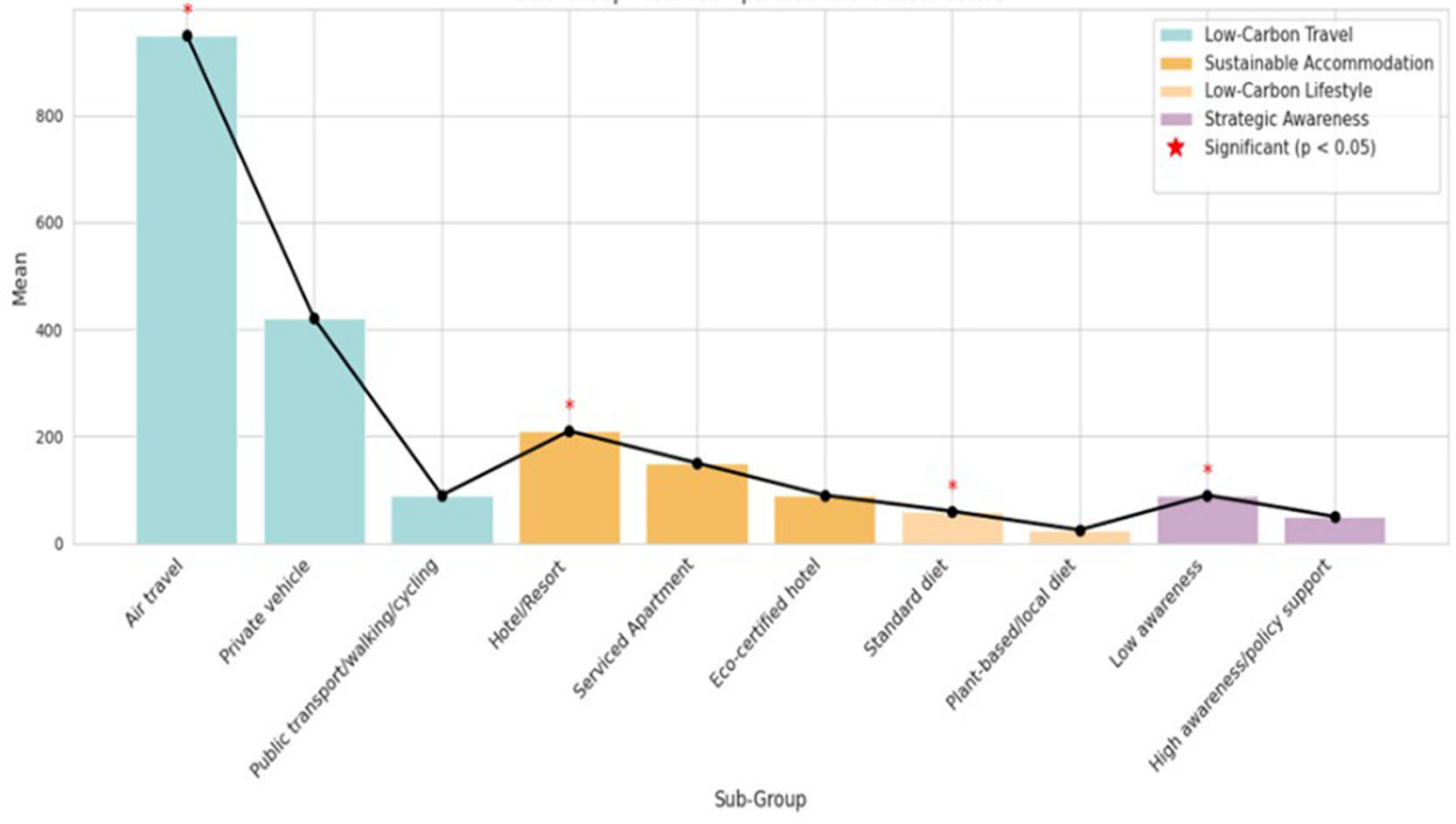
One-Way ANOVA–based comparison of carbon emissions in longevity tourism.

According to the One-Way ANOVA findings in Table 5 and Figure 4, there are significant differences in the amount of carbon emissions among the essential travel factors, such as lodging, transport, food preference, and strategic awareness $\left(p<0.01\right).$ Public transportation, eco-certified housing, and plant-based diets generated the lowest emissions, while air travel and traditional hotels had the highest. This indicates opportunities for implementing low-carbon strategies in longevity tourism.

**Table 5.** One-Way ANOVA of carbon emissions by key travel factors (n = 450).

Main theme	Sub-group	Mean ( *kg* *CO*_2_ *e*)	*F−value*	*p−value*	Significance
**Low-Carbon Travel Behavior**	Air travel	950	28.45	<0.001	Significant
	Private vehicle	420			
	Public transport/walking/cycling	90			
**Sustainable Accommodation Choices**	Hotel/Resort	210	19.32	<0.001	Significant
	Serviced Apartment	150			
	Eco-certified hotel	90			
**Low-Carbon Lifestyle Adoption**	Standard diet	60	12.87	<0.001	Significant
	Plant-based/local diet	25			
**Strategic Awareness and Support**	Low awareness	90	10.12	0.006	Significant
	High awareness/policy support	50			

Note. F-values and p-values are reported for each one-way ANOVA model. Degrees of freedom are reported as F (df_between, df_within). η ^2^ = partial eta-squared (effect size). Post-hoc: Tukey HSD applied for Low-Carbon Travel Behavior (3 groups, homogeneous variance confirmed by Levene's test, p = .312) and Sustainable Accommodation Choices (3 groups, Levene's p = .184); Welch's F and Games-Howell used for Low-Carbon Lifestyle Adoption and Strategic Awareness and Support where Levene's test indicated heteroscedasticity (p < .05). All pairwise group differences reported in the text above are significant at p < .05.

For Low-Carbon Travel Behavior (transport mode), the one-way ANOVA revealed a statistically significant effect (F(2, 447) = 28.45, p < .001, η ^2^ = .113), indicating a large effect; post-hoc comparisons (Tukey HSD) confirmed that all three transport mode groups differed significantly from one another (all pairwise p < .001). For Sustainable Accommodation Choices, a significant effect was found (F(2, 447) = 19.32, p < .001, η ^2^ = .079, moderate effect); eco-certified hotel guests emitted significantly less than both hotel/resort guests (p < .001) and serviced apartment guests (p = .003). For Low-Carbon Lifestyle Adoption, the ANOVA was significant (F(1, 448) = 12.87, p < .001, η ^2^ = .028, small-to-moderate effect); plant-based/local diet tourists emitted significantly less than standard diet tourists (p < .001). For Strategic Awareness and Support, the effect was also significant (F(1, 448) = 10.12, p = .006, η ^2^ = .022); high-awareness tourists emitted significantly less than low-awareness tourists (p = .006). Public transportation, eco-certified housing, and plant-based diets generated the lowest emissions, while air travel and traditional hotels had the highest. This indicates opportunities for implementing low-carbon strategies in longevity tourism.

### 4.5 Thematic analysis

**Figure 5.**
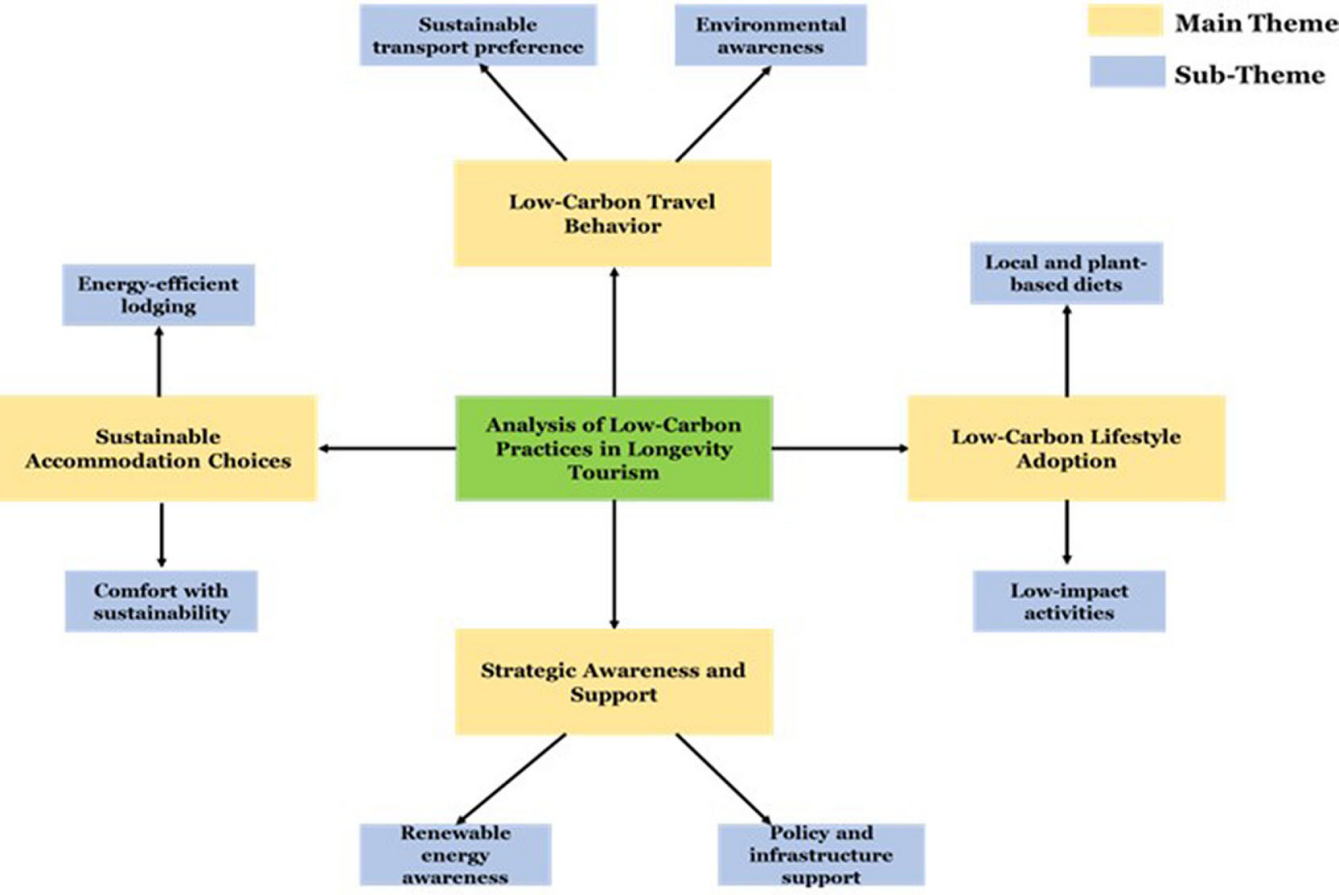
Thematic framework of low-carbon practices in longevity tourism in Thailand.

The thematic analysis shows that the low-carbon travelling behavior such as the eco-friendly living that is reported in Table 6 and Figure 5 is being adopted by longevity travellers. The knowledge and availability of renewable energy in the region enhances travel satisfaction and destination loyalty. These insights underscore the significance of intentional low-carbon management to reduce emissions while maintaining Thailand’s appeal as a long-term vacation spot.

**Table 6.** Thematic analysis of low-carbon practices in longevity tourism.

Main theme	Sub-theme	Short description
**Low-Carbon Travel Behavior**	Sustainable transport preference	Tourists preferred public transport, walking, and cycling to reduce travel-related emissions.
	Environmental awareness	Transport choices were influenced by concern for carbon reduction.
**Sustainable Accommodation Choices**	Energy-efficient lodging	Preference for eco-certified and energy-saving accommodations during long stays.
	Comfort with sustainability	Visitors valued comfort and wellness alongside low-carbon features.
**Low-Carbon Lifestyle Adoption**	Local and plant-based diets	Adoption of low-carbon food choices for health and environmental benefits.
	Low-impact activities	Interest in walking tours and locally organized, low-emission activities.
**Strategic Awareness and Support**	Renewable energy awareness	Awareness of renewable energy use enhanced destination image and trust.
	Policy and infrastructure support	Demand for better low-carbon infrastructure and information support.

The following illustrative participant quotes provide direct evidence for each thematic finding:

Low-Carbon Travel Behavior (Sustainable Transport Preference): “I stopped renting cars completely after the first week. Walking and songthaew [local shared taxis] are actually faster in the old city, and I realized I wasn’t adding to the pollution that bothers my breathing anyway” (International participant, Chiang Mai, aged 67).

Sustainable Accommodation Choices (Energy-Efficient Lodging): “When I found the hotel had solar water heating and recycled rainwater for the gardens, it made me feel the extra cost was worth it. I knew my long stay wasn’t hurting the place” (International participant, Hua Hin, aged 71).

Low-Carbon Lifestyle Adoption (Local and Plant-Based Diets): “Thai food is naturally lighter and more plant-based than what I eat at home. I genuinely eat better here and I know it’s better for the environment too — so it’s not a sacrifice, it’s a pleasure” (Domestic participant, Bangkok, aged 58).

Strategic Awareness and Support (Policy and Infrastructure): “If the buses came more frequently and the cycle paths connected the wellness center to the beach, I would never need a taxi at all. The infrastructure just isn’t quite there yet, but I can see they are trying” (International participant, Phuket, aged 63).

## 5. Discussion

Current low-carbon tourism and carbon footprint studies have yielded results that are not as extensive because they have a variety of limitations. The high level of reliance on self-reported data on travel activities and focus on specific geographic locations can introduce response bias and make findings not as generalizable to other tourism settings ( Janchai & Suvittawat, 2025). Moreover, it is constrained by the nature of the SEM due to its common usage in SEM which generally provides descriptive information only ( Tong et al., 2022). Moreover, it needs to be cross-cultural and multi-regional to ensure it is applicable to more than just domestic tourism within any one country such as Thailand as research carried out in a single country such as Thailand cannot give the true picture of the travelling habits and emission patterns of the international longevity tourists ( Fakfare & Wattanacharoensil, 2024; Guo et al., 2025).

The ANOVA models in this study compared emission levels across behavioral sub-groups (transport mode, accommodation type, dietary pattern, and awareness level) without adjusting for potentially confounding variables. Length of stay is the most consequential of these: longer-staying visitors accumulate greater absolute emissions in accommodation and lifestyle domains even if their per-day emissions are lower, and the positive association between stay duration and sustainable accommodation choice observed in the descriptive data ( Table 2 shows 79.5% of respondents staying more than one month) may partially account for the lower per-capita emissions in eco-certified accommodation. Similarly, international visitors (59.6% of the sample) arrive predominantly by long-haul air, inflating transportation emissions relative to domestic visitors, and age may correlate with wellness activity participation rates and therefore with lifestyle emission levels. While the purpose of this study was to describe and compare emission magnitudes across behavioral categories rather than to model causal determinants, future research should employ ANCOVA or multiple regression models that adjust for length of stay, nationality, and age as covariates when estimating the independent effect of accommodation type, transport mode, and behavioral awareness on per-capita emissions. The current findings should therefore be interpreted as association-based estimates of emission differences across behavioral groups, subject to the confounding effects noted above.

The research offers detailed, context-sensitive information going beyond the classical low-carbon tourist research through assessing longevity tourism in Thailand through an LCA-based carbon footprint model. It integrates both statistical analysis and process-based emission accounting to show how the long-term travel patterns, accommodation choices and mode of transportation lead to measurable carbon outputs. This form of synergy reduces the impact of the longevity tourism on the environment without having an impact on the wellbeing of the tourists and can strategize the low carbon travel and provides a model that can be tested and altered in other geographic and cultural locations ( Raihan, 2024a, 2024b).

The findings provide a directly evidence-grounded basis for each component of the proposed strategic management framework for low-carbon longevity tourism. First, the strategic priority of low-carbon transport infrastructure — including improved public transport connectivity and cycling infrastructure at longevity destinations — is directly supported by the ANOVA finding that transport is the single largest emission domain (mean 347 kg CO _2_e; F(2, 447) = 28.45, p < .001, η ^2^ = .113) and by the qualitative theme that infrastructure access, rather than willingness, is the primary barrier to sustainable transport adoption among longevity tourists (e.g., “if the buses came more frequently and the cycle paths connected the wellness center to the beach, I would never need a taxi”). Second, the priority of expanding eco-certified accommodation supply is supported by the finding that eco-certified guests produce less than half the accommodation emissions of hotel/resort guests (90 vs. 210 kg CO _2_e; F(2, 447) = 19.32, p < .001, η ^2^ = .079), and by the qualitative finding that knowledge of eco-certification increases perceived destination value and loyalty among long-stay visitors. Third, the recommendation to promote local and plant-based dietary options at wellness facilities is grounded in the finding that plant-based/local diet consumers produce less than half the dietary emissions of standard diet consumers (25 vs. 60 kg CO _2_e; F(1, 448) = 12.87, p < .001, η ^2^ = .028) and in the qualitative observation that Thai cuisine’s natural plant-orientation makes this a low-resistance behavioral shift. Fourth, the recommendation to invest in renewable energy infrastructure and carbon-awareness programming is supported by the finding that high-awareness tourists produce lower emissions across all domains (strategic awareness ANOVA: F(1, 448) = 10.12, p = .006, η ^2^ = .022) and by qualitative evidence that renewable energy visibility enhances destination image and trust. Together, these evidence-to-recommendation linkages demonstrate that the proposed framework is grounded in the specific empirical findings of this study.

## 6. Conclusion

To estimate the carbon footprint of longevity tourism in Thailand and give strategic suggestions on how to promote low-carbon tourism and retain the satisfaction of tourists. The research employed a mixed method design to collect survey data on 450 longevity visitors and applied one-way ANOVA, thematic analysis, descriptive statistics and carbon footprint analysis based on the LCA. The average per-capita carbon footprint according to the results was 582 kg CO2e, housing (160 kg CO2e) and transportation (347 kg CO2e) were the highest contributors. The extensive differences in emissions were observed in the type of housing, activity patterns, food habits, and the choice of the mode of transportation $\left(p<0.001\right).$ Thematic analysis indicates that low-carbon practices, green lodging, veganism, low-impact activities, and green energy awareness significantly influence travel decisions. As this study employs a cross-sectional, observational design, the following findings describe associations and patterns rather than causal effects. Conclusions are most directly applicable to longevity tourists at the five studied Thai destinations and should be extrapolated to other contexts with appropriate caution. These findings suggest that deliberate low-carbon interventions, optimized infrastructure, and behavioral support programs are promising strategies for enhancing the sustainability of longevity tourism, though the causal mechanisms underlying these associations should be confirmed through longitudinal and experimental designs. To enhance sustainable tourism, deliberate low-carbon interventions, optimized infrastructure, and behavioral coaching are essential.

### Limitation and future work

This study has a few limitations that impact the generalizability of its findings. First, data were collected at five Thai destinations, which, while selected to represent the diversity of longevity tourism in Thailand, do not capture all destination types or regional contexts within the country. Findings should be considered most directly applicable to established longevity tourism hubs in Thailand with developed wellness infrastructure. Second, the study focuses exclusively on the longevity tourist segment as defined in this paper; the emission patterns and behavioral dynamics identified here may not be directly transferable to short-stay leisure tourists, backpackers, medical tourists without wellness components, or longevity tourists in other national contexts with different transport networks, energy grids, or dietary cultures. Third, as noted in the Discussion, the ANOVA analyses do not control for potential confounders such as length of stay, age, and nationality. Fourth, the reliance on self-reported travel activity data introduces the possibility of recall and social desirability bias in emission estimates. These limitations point to several priorities for future research: longitudinal studies tracking the same longevity tourists across multiple visits to assess whether low-carbon behaviors are sustained; comparative studies across multiple destination countries to establish cross-cultural generalizability; multivariate regression analyses controlling for confounders; and intervention-based studies testing the effectiveness of specific low-carbon infrastructure investments and behavioral nudges in longevity tourism contexts.

## Data Availability

The anonymized primary dataset underpinning this study — including de-identified survey questionnaire responses, travel activity log data (with all direct personal identifiers removed), derived per-capita carbon emission estimates, and the emission factor reference table used in LCA calculations — has been deposited in the Figshare repository. Madhyamapurush, Warach (2026). Strategic Management of Low Carbon Travel in Longevity Tourism Evidence from Thailand. figshare. Dataset. https://doi.org/10.6084/m9.figshare.31362268.v2 ( Madhyamapurush, Warach, 2026). Data are available under the terms of the Creative Commons Attribution 4.0 International license (CC-BY 4.0).
