# Supplementary material for: Strategic Management of Low Carbon Travel in Longevity Tourism Evidence from Thailand
Source: F1000Res. 2026 Apr 24;15:404. Originally published 2026 Mar 17. [Version 2] doi: 10.12688/f1000research.178615.2 (PMC13139847; doi:10.12688/f1000research.178615.2)
Supplement: Peer review history

# Reviewer report: Reviewer response for version 2

**DOI:** 10.5256/f1000research.199121.r478882

**Published:** 2026-05-04

**Authors:** I Gusti Bagus Rai Utama

**Affiliations:** 1 Universitas Dhyana Pura, Bali, Indonesia

The paper has been accepted for indexing following revisions that adequately addressed the reviewers’ comments.

 This revised version incorporates substantial methodological, analytical, and reporting enhancements. The most significant changes are as follows: a formal working definition of longevity tourism has been introduced in the Introduction, specifying the age threshold, minimum length of stay, and primary travel motivations, along with clear differentiation from related constructs such as wellness tourism and retirement migration. A dedicated research gap subsection has also been added to the literature review to more precisely position the study within the existing body of knowledge.

 The Methods section has been significantly expanded. A new sampling subsection outlines the purposive convenience sampling strategy, including the five destination sites with their selection rationale, as well as the inclusion and exclusion criteria and response rate. An operational definition of longevity tourists as applied in the field has been provided. In addition, questionnaire validation procedures, Cronbach’s alpha values for all four construct domains, and a detailed description of the thematic analysis procedure based on Braun and Clarke’s (2006) framework have been incorporated. The LCA methodology subsection now includes detailed information on system boundaries, emission factor sourcing, per capita calculation approaches, and sensitivity analysis procedures.

 Statistical reporting has been strengthened throughout the manuscript. Complete ANOVA results — including F values, degrees of freedom, exact p-values, partial eta-squared effect sizes, and post hoc comparisons — are now presented for all four models, along with assumption testing procedures and Levene’s test results. Standard deviations and 95% confidence intervals have also been added to the descriptive statistics tables.

 The Discussion and Conclusions sections have been revised to replace causal language with association-based interpretations appropriate for a cross-sectional observational design. Furthermore, a Limitations and Future Work subsection has been included, along with a formal Data Availability Statement providing access via a Figshare repository link

Is the work clearly and accurately presented and does it cite the current literature?

Partly

If applicable, is the statistical analysis and its interpretation appropriate?

Partly

Are all the source data underlying the results available to ensure full reproducibility?

Partly

Is the study design appropriate and is the work technically sound?

Partly

Are the conclusions drawn adequately supported by the results?

Partly

Are sufficient details of methods and analysis provided to allow replication by others?

Partly

Reviewer Expertise:

NA

I confirm that I have read this submission and believe that I have an appropriate level of expertise to confirm that it is of an acceptable scientific standard.

# Reviewer report: Reviewer response for version 1

**DOI:** 10.5256/f1000research.197024.r468643

**Published:** 2026-04-07

**Authors:** Anila Thomas

**Affiliations:** 1 Jyoti Nivas College Autonomous, Bengaluru, Karnataka, India

An insightful and pertinent analysis of the environmental effects of longevity tourism can be found in the article "Strategic Management of Low Carbon Travel in Longevity Tourism: Evidence from Thailand." The study effectively combines tourist management with sustainability issues, with a special emphasis on carbon emissions and low-carbon travel tactics.

 The study is strong, with a good sample and insightful conclusions about carbon emissions in long-term travel. The grammar, scholarly review, methodology specifications, questionnaire validation, easy access to data, and feasibility appraisal also need some substantial modifications. Despite being precise, the literature review lacks more detailed evaluation and theoretical framework and is descriptive rather than analytical in nature. When examining variances in emissions between groups, descriptive statistics and One-Way ANOVA are recommended.

**Final Recommendation**

**Suggestions Modifications**:

- Enhance the manuscript's academic writing style and cognitive quality throughout.
- To guarantee consistent quality, include more thorough methodological explanations.
- Critical analysis and more comprehensive conceptual framework will strengthen the literature evaluation.
- Increase openness about LCA parameters and data being accessible.
- Extend the conversation to offer more in-depth explanations of the findings' consequences.

**Approved with Recommended Revisions**

 The manuscript presents a valuable contribution to the field of sustainable tourism, particularly in the context of longevity tourism and low-carbon travel strategies. But it requires revisions in clarity, methodological transparency, and data reporting before it can be considered fully sound to be indexed.

Is the work clearly and accurately presented and does it cite the current literature?

Yes

If applicable, is the statistical analysis and its interpretation appropriate?

Yes

Are all the source data underlying the results available to ensure full reproducibility?

Yes

Is the study design appropriate and is the work technically sound?

Yes

Are the conclusions drawn adequately supported by the results?

Yes

Are sufficient details of methods and analysis provided to allow replication by others?

Yes

Reviewer Expertise:

My research interests include the Sustainable Development Goals (SDGs) and Tourism Destinations, Resource Sustainability, AI Applications in the Tourism and Hospitality Industry, Geographic Relevance to  Tourist Destination Development, Health and Wellness Tourism, Women's Participation in Ayurvedic Medical Tourism, and Community Involvement in Tourism Resource Preservation.

I confirm that I have read this submission and believe that I have an appropriate level of expertise to confirm that it is of an acceptable scientific standard.

# Reviewer report: Reviewer response for version 1

**DOI:** 10.5256/f1000research.197024.r468642

**Published:** 2026-03-28

**Authors:** I Gusti Bagus Rai Utama

**Affiliations:** 1 Universitas Dhyana Pura, Bali, Indonesia

**Assessment: Partly**

 Strengths:

- The article follows a clear structure (introduction, related works, methods, results, implications, conclusion), and tables summarizing demographics and emission estimates are useful and readable.​
- The abstract and introduction effectively communicate the motivation and key findings, including the dominance of transport and accommodation in emissions and the rationale for focusing on longevity tourism in Thailand.​
- Recent literature (2021–2025) on tourism emissions, low‑carbon policies, and related topics is cited, indicating engagement with current work.​

Issues and recommendations (to address):

1. **Conceptual clarity of “longevity tourism” (must address):**
  - The term is used but not precisely defined or consistently linked to related concepts such as long-stay tourism, wellness tourism, or retirement migration.​
  - Recommendation: Provide a clear definition early in the introduction, including age thresholds, typical length of stay, main purposes (wellness, retirement, etc.), and how this concept aligns or differs from existing constructs in the tourism literature.
2. **Articulation of the research gap (should address):**
  - The related works section is extensive but largely descriptive; it discusses many country‑specific studies without clearly synthesizing the gap this study fills beyond adding a Thai case.​
  - Recommendation: Conclude the literature review with a concise statement of what is missing in current research (e.g., integrated LCA-based assessment for longevity tourism specifically, behavior-sensitive frameworks in Thailand) and how this study addresses that gap.
3. **Consistency and completeness of citations (should address):**
  - Several citations (especially 2025 and 2024 references) are mentioned in the text; their completeness and consistency in the reference list should be checked.​
  - Recommendation: Ensure all in‑text citations appear in the reference list with full details, and that citation style is consistent.

Study design and technical soundness

**Assessment: Partly**

 Strengths:

- The mixed‑method design (survey + activity logs + national emission factors + LCA + ANOVA + thematic analysis) is appropriate for the goals of estimating emissions and exploring associated behaviors and perceptions in longevity tourism.​
- The sample size (450 respondents) is adequate for the planned analyses, and demographic details (Table 2) provide a useful profile of the respondents.​

Issues and recommendations (to address):

1. **Sampling and recruitment (must address):**
  - The paper does not clearly describe how participants were selected (e.g., sampling frame, recruitment sites, random vs convenience sampling, inclusion/exclusion criteria, response rate).​
  - Recommendation: Add a dedicated subsection describing sampling strategy, recruitment locations (e.g., specific destinations, types of facilities), criteria for defining and including “longevity tourists,” and potential sources of sampling bias.
2. **Operational definition of “longevity tourists” (must address):**
  - Although the sample is described as older, long‑stay visitors, the explicit criteria used to categorize someone as a “longevity tourist” are not stated.​
  - Recommendation: Define “longevity tourists” operationally (e.g., age threshold, minimum length of stay, motivation for travel) and explain how this definition was applied in the field.
3. **Description of study sites (should address):**
  - The phrase “selected Thai destinations” is vague; no details are given about the types of destinations (urban, rural, coastal, wellness hubs), which affects generalizability.​
  - Recommendation: Briefly describe the destinations included (e.g., major tourism regions, types of facilities) and justify why they are representative (or not) of longevity tourism in Thailand.
4. **Integration of mixed methods (should address):**
  - While both quantitative and qualitative components are mentioned, the paper does not explain how they are integrated (e.g., whether qualitative findings were used to interpret quantitative results or to refine the framework).​
  - Recommendation: Clarify the mixed‑method design type (e.g., explanatory sequential) and describe how qualitative themes complemented or enriched the quantitative findings.

Methods and analysis detail for replication

**Assessment: Partly**

 Strengths:

- The main analytical procedures are named: use of national databases for emission factors, LCA‑based carbon footprint analysis, descriptive statistics and one‑way ANOVA in SPSS, and thematic analysis.​
- Table 1 provides the core questionnaire items for four conceptual domains (Low‑Carbon Travel Behavior, Sustainable Accommodation Choices, Low‑Carbon Lifestyle Adoption, Strategic Awareness and Support).​

Issues and recommendations (to address):

1. **Questionnaire and measurement (must address):**
  - Only selected items are shown; response scales, exact wording of all items, and any pre‑testing or validation are not reported.​
  - Recommendation:
    - Provide the full questionnaire as supplementary material, including response options (Likert scales, yes/no, frequency categories) and any screening questions.
    - Report basic psychometrics of the constructs (e.g., internal consistency, any factor analysis) if composite indices are used.
2. **LCA and emission factor specification (must address):**
  - The LCA methodology is described in general terms only; specific emission factors, data sources (names of databases, years), system boundaries, and calculation formulae are not detailed.​
  - Recommendation:
    - Identify all emission factor sources (e.g., specific national inventory or tourism datasets, publication years).
    - Clearly state system boundaries (direct emissions, upstream energy production, infrastructure, etc.).
    - Provide formulae showing how per‑capita emissions were calculated for transport, accommodation, diet/activities, and awareness/infrastructure, including assumptions about length of stay, trip frequency, and load factors.
3. **Variable construction (should address):**
  - It is unclear how construct scores such as “Low‑Carbon Travel Behavior” or “Sustainable Accommodation Choices” are derived from the questionnaire items, and how these are linked to emission estimates.​
  - Recommendation: Explain coding and aggregation (e.g., summing or averaging Likert items, converting yes/no responses to numerical scores), and clarify how these variables are used in the ANOVA models.
4. **Thematic analysis procedures (should address):**
  - Thematic analysis is mentioned but not described in operational terms (coding process, number of coders, reliability).​
  - Recommendation: Outline the qualitative analysis steps (coding framework, inductive/deductive approach), whether multiple coders were involved, how disagreements were resolved, and provide illustrative quotes for key themes in the results or supplementary material.

Statistical analysis and interpretation

**Assessment: Partly**

 Strengths:

- The use of descriptive statistics and one‑way ANOVA to examine differences in emissions across transport modes, accommodation types, lifestyle patterns, and awareness levels is reasonable given the aims.​
- The paper conveys the substantive conclusion that transportation and accommodation produce much higher per‑capita emissions than lifestyle and awareness factors, which is consistent with the reported averages in Table 3.​

Issues and recommendations (to address):

1. **Incomplete ANOVA reporting (must address):**
  - Only significance at p < 0.01 is mentioned; F‑statistics, degrees of freedom, exact p‑values, and effect sizes are not reported, nor are post‑hoc comparisons.​
  - Recommendation: For each ANOVA, report F, df, p‑values, and effect sizes (e.g., eta‑squared), specify which dependent and independent variables were used, and describe any post‑hoc tests and which groups differed significantly.
2. **Assumptions and robustness (should address):**
  - There is no mention of checking ANOVA assumptions (normality, homogeneity of variance) or steps taken if they were violated.​
  - Recommendation: Indicate how assumptions were assessed (e.g., residual plots, Levene’s test) and whether transformations or robust methods were needed.
3. **Potential confounders and multivariate analysis (should address):**
  - Important factors such as length of stay, age, and nationality could influence emission levels, but appear only descriptively, not as controls in the analyses.​
  - Recommendation: At minimum, discuss the potential confounding effects of these variables; ideally, consider multivariate models (e.g., regression or ANCOVA) that adjust for these factors when estimating differences in emissions across modes and accommodation types.
4. **Measures of variability and uncertainty (should address):**
  - The paper focuses on means; standard deviations, confidence intervals, or other uncertainty measures for key emission estimates are not clearly presented.​
  - Recommendation: Add variability measures to tables and, if feasible, conduct sensitivity analyses for key assumptions (e.g., alternative emission factors or stay lengths).

Source data and reproducibility

**Assessment: Partly**

 Strengths:

- The manuscript clearly identifies the key data sources (survey, activity logs, national databases) and shows aggregate results in tables and figures.​

Issues and recommendations (to address):

1. **Data and code availability statement (must address):**
  - There is no explicit statement that de‑identified survey data, activity logs, and derived emission datasets are publicly available, nor any repository links.​
  - Recommendation: Deposit anonymized data (with a codebook) and, where feasible, analysis scripts or SPSS syntax in a suitable repository, and include a formal data availability statement with accession details.
2. **Emission factor transparency (should address):**
  - The specific emission factor tables used from national databases are not accessible in the paper.​
  - Recommendation: Either reproduce key emission factors in an appendix or supplementary file, or provide clear references and URLs to the exact versions used.

Conclusions and support from results

**Assessment: Partly**

 Strengths:

- The empirical findings that transportation and accommodation dominate emissions, while lifestyle and awareness factors contribute less but still matter, are consistent with the LCA-based results (347 kg CO2e for transport, 160 for accommodation, versus 42.5 and 50 for lifestyle and awareness, respectively).​
- The proposed focus on low‑carbon planning, infrastructure, and energy‑efficient lodging logically follows from the evidence that these domains are key emission sources.​

Issues and recommendations (to address):

1. **Causal language vs observational design (must address):**
  - Some conclusions imply causal effects of certain policies or frameworks, even though the study is cross-sectional and observational.​
  - Recommendation: Rephrase claims to emphasize association and potential rather than demonstrated causal impact (e.g., “findings suggest that…” rather than “X leads to…”).
2. **Scope and generalizability (should address):**
  - The generalization of results beyond the sampled destinations and specific longevity tourist segment is not sufficiently qualified.​
  - Recommendation: Explicitly acknowledge the geographical and sample limitations and clarify that conclusions are most directly applicable to the studied contexts in Thailand.
3. **Linking recommendations to evidence (should address):**
  - While the proposed strategic framework is plausible, some policy recommendations are not explicitly tied to particular quantitative or qualitative findings in the study.​
  - Recommendation: Strengthen the discussion by linking each major policy recommendation to specific empirical results or themes identified in the data.

Points that must be addressed for scientific soundness

 To make the article scientifically sound and fully consistent with F1000Research standards, the following **must** be addressed:

1. Provide a clear operational definition of “longevity tourists” and describe the sampling and recruitment procedures (including study sites and inclusion criteria).​
2. Expand the methods section to fully document the questionnaire (with scales and, if possible, validation), the LCA methodology (emission factors, system boundaries, calculation details), and the construction of key variables.​
3. Improve statistical reporting for the ANOVA analyses, including full results (F, df, p, effect sizes), assumptions, and post‑hoc tests.​
4. Add a formal data availability statement and make de‑identified data and, where feasible, analysis code publicly accessible.​
5. Moderate causal language in the conclusions and explicitly acknowledge the observational nature of the study and its contextual limitations.​

Once these core issues are addressed, the paper will provide a more solid and reproducible contribution to the emerging literature on low‑carbon management in longevity tourism.

Is the work clearly and accurately presented and does it cite the current literature?

Partly

If applicable, is the statistical analysis and its interpretation appropriate?

Partly

Are all the source data underlying the results available to ensure full reproducibility?

Partly

Is the study design appropriate and is the work technically sound?

Partly

Are the conclusions drawn adequately supported by the results?

Partly

Are sufficient details of methods and analysis provided to allow replication by others?

Partly

Reviewer Expertise:

Tourism Management

I confirm that I have read this submission and believe that I have an appropriate level of expertise to confirm that it is of an acceptable scientific standard, however I have significant reservations, as outlined above.
